# Eco-microbiology: discovering biochemical enhancers of PET biodegradation by *Piscinibacter sakaiensis*

**DOI:** 10.1128/aem.02118-24

**Published:** 2025-02-24

**Authors:** Felipe-Andrés Piedra, Miguel A. Salazar, Sara Abouelniaj, Raayed Rahman, Justin C. Clark, Yimo Han, Zhao Wang, Anthony Maresso

**Affiliations:** 1 Department of Molecular Virology and Microbiology, Baylor College of Medicine 189531 , Houston, Texas, USA; 2 Department of Materials Science and NanoEngineering, Rice University 539850 , Houston, Texas, USA; 3 Verna and Marrs McLean Department of Biochemistry and Molecular Pharmacology, Baylor College of Medicine 3989 , Houston, Texas, USA; 4 Department of Molecular and Cellular Biology, Baylor College of Medicine 3989 , Houston, Texas, USA; 5 Department of Molecular and Cellular Oncology, Division of Basic Science, The University of Texas MD Anderson Cancer Center 4002 , Houston, Texas, USA; Shanghai Jiao Tong University, Shanghai, China

**Keywords:** eco-microbiology, bioremediation, plastic pollution, biodegradation, fermentation, *Piscinibacter sakaiensis*, PET, environmental microbiology, extrinsic biochemical stimulation

## Abstract

The scale of plastic pollution boggles the mind. Nearly 400 megatons of virgin plastics are produced annually, with an environmental release rate of 80%, and plastic waste, including microplastics and nanoplastics, is associated with a plethora of problems. The naturally evolved abilities of plastic-degrading microbes offer a starting point for generating sustainable and eco-centric solutions to plastic pollution—a field of endeavor we term eco-microbiology. Here, we developed an iterative discovery procedure coupling faster polyethylene terephthalate (PET)-dependent bioactivity screens with longer-term PET biodegradation assays to find biochemical boosters of PET consumption by the bacterium *Piscinibacter sakaiensis*. We discovered multiple hits supporting the enhancement of PET biodegradation, with a 0.39% dilution of growth medium #802, a rich medium similar to Luria-Bertani broth, on average more than doubling the rate of PET biodegradation both alone and in combination with 0.125% ethylene glycol. In addition, we identified other chemical species (sodium phosphate, L-serine, GABA) worth further exploring, especially in combination with growth medium #802, for enhanced PET biodegradation by *P. sakaiensis*. This work represents an important step toward the creation of a low-cost PET fermentation process needed to help solve PET plastic pollution.

**IMPORTANCE:**

Plastic pollution is an urgent issue. Adding to the well-known problems of bulk plastic litter, shed microplastics and nanoplastics are globally distributed, found in diverse organisms including human foodstuffs and tissues, and increasingly associated with chronic disease. Solutions are needed and the microbial world offers abundant help via naturally evolved consumers of plastic waste. We are working to accelerate polyethylene terephthalate (PET) plastic biodegradation by *Piscinibacter sakaiensis*, a recently described bacterium that evolved to slowly but completely consume PET, one of the most common types of plastic pollution. We used a combination of PET-dependent bioactivity screens and biodegradation tests to find stimulators of PET biodegradation. Out of hundreds, we found a small number of biochemical conditions that more than double the PET biodegradation rate. Our work provides a foundation for further studies to realize a fermentation process needed to help solve PET plastic pollution.

## INTRODUCTION

Plastic remains a revolutionary class of materials, offering an extraordinary range and combination of desirable properties. However, its durability combined with a massive and accelerating scale of production means that plastic pollution has become a global and rapidly growing problem (1–3).

In its roughly seven decades of existence, over 8 gigatons of plastic materials have been released into the global environment, and the current annual rate of release is approximately 0.3 gigatons and growing (1). Bulk plastic pollution creates a series of problems: as a sign of community and environmental neglect, and by virtue of its association with runaway climate change, it contributes to a widespread feeling of dismay (4); it can cause a plethora of infrastructure issues, especially in water management and flood control (5, 6); and it can be straightforwardly problematic to wildlife, causing injury or death to large organisms, especially marine life, via physical capture or accidental ingestion. In addition, bulk plastic materials weather and degrade into smaller debris and microplastics and nanoplastics (MNPs). It is now well established that MNPs, like other forms of small particulate matter, circulate globally through a variety of geophysical processes including atmospheric transport through the free troposphere (7, 8). MNPs also readily enter a staggering variety of organisms and tissues, including some of the most common fruits and vegetables consumed by humans and both male and female human reproductive organs (7, 9–13). Once within an organism, a variety of pathologies become possible, whether due to the materials themselves (ex: contributing to the blockage of small arteries) or by virtue of the ability of MNPs to adsorb and deliver other chemicals including a plethora of plastic additives of varying toxicity (3, 7, 11, 13–17).

Addressing plastic pollution is therefore of vital concern, and it should not be surprising that a variety of microbes have already started (18, 19). Indeed, all plastics are polymeric hydrocarbons that can serve as a carbon source for microbes able to degrade them, notwithstanding other well-known (e.g., substrate for biofilm formation [20–22]) and potential microbial uses. The major issue remains the scale and speed of the generation of plastic pollution, which demands both the adoption of sustainable patterns of production and consumption and cleaning up at a planetary scale. For the latter, the naturally evolved abilities of plastic-degrading microbes can be tapped to create low-cost technologies for the rapid and widespread biodegradation of plastic waste.

One such microbe is the recently described aerobic gram-negative bacterium *Piscinibacter sakaiensis* (19). *P. sakaiensis* evolved to degrade and completely consume the plastic polyethylene terephthalate (PET), a semi-aromatic polyester abundantly used in textiles, clothing, and food and beverage packaging. PET is currently produced at over 50 megatons per year, and over 50% is environmentally released. PET-based food and beverage packaging is among the most common and eye-catching forms of bulk plastic pollution, and PET MNPs are some of the most common in human tissues (12, 23, 24). *P. sakaiensis* degrades PET into soluble fragments and further into its basic chemical building blocks of ethylene glycol (EG) and terephthalic acid (TA), which are further catabolized and completely incorporated into the organism itself, either as basic biochemical constituents (nucleic and amino acids, lipids, etc.) or into another polymeric form, polyhydroxyalkanoate (PHA), which is presumably used as a carbon/energy source by *P. sakaiensis* when needed (19, 25, 26). PHAs are readily degraded and consumed by other microbes (27).

The first and most important step in the biodegradation of PET by *P. sakaiensis* is accomplished by a single secreted enzyme, the thermolabile IsPETase, which cleaves the ester bonds of single PET chains into monoethylene terephthalate (MHET) along with trace amounts of bisethylene glycol terephthalate (BHET) and terephthalic acid (19). MHET is further degraded by a periplasmic MHETase (19, 28). Strikingly, the IsPETase works at temperatures (RT to ca. 30°C) considerably lower than those previously thought necessary for PET biodegradation (29–31). Indeed, the first PET-hydrolyzing enzymes discovered were thermostable cutinases and esterases able to operate at temperatures near the PET glass transition (ca. 65–80°C) where substantial PET chain mobility in amorphous to low crystallinity materials should make single PET chains more vulnerable to enzymatic attack (30–33). Significant progress has been made using mutated versions of the thermostable leaf compost cutinase, including the development of an industrial-scale procedure for rapid degradation of highly processed PET waste (34, 35). Nevertheless, the considerable activity shown by IsPETase at lower temperatures has made it the subject of intense study in recent years. Its activity and stability have been greatly improved via a combination of rational and more agnostic mutagenesis strategies (36–39). However, in contrast to the surge in enzymology provoked by its star enzyme, *P. sakaiensis* has been relatively ignored since the publication of its discovery, and little to no effort has been made to improve its extraordinary but quantitatively modest ability (~0.2 mg cm^−2^ day^−1^) to completely consume amorphous to low crystallinity PET under exceedingly mild conditions, yielding only biomass, CO_2_, and water as byproducts.

We endeavored to begin bridging this gap by searching chemical/nutrient space for molecules, and combinations and mixtures thereof, that stimulate enhanced PET biodegradation by *P. sakaiensis*. Such extrinsic factors could stimulate PET-dependent metabolism and/or gene expression or even enhance the surface properties of PET related to its degradability. This approach to finding a culture medium more supportive of PET biodegradation by *P. sakaiensis* is one step in a larger strategy toward the development of a low-cost fermentation-based procedure for the rapid conversion of PET waste to microbial biomass.

## RESULTS

### Phenotype microarray screening for enhanced PET-dependent bioactivity

Bacteria are highly adaptable, combining the constant sensing of environmental stimuli including discrete chemical cues with manifold metabolic and gene regulatory responses. This adaptability can be exploited experimentally and tuned toward a desired behavior. Thus, we hypothesized the existence and discoverability of small molecules and ionic compounds able to upregulate the naturally evolved propensity of *P. sakaiensis* to consume PET.

**Fig 1:**
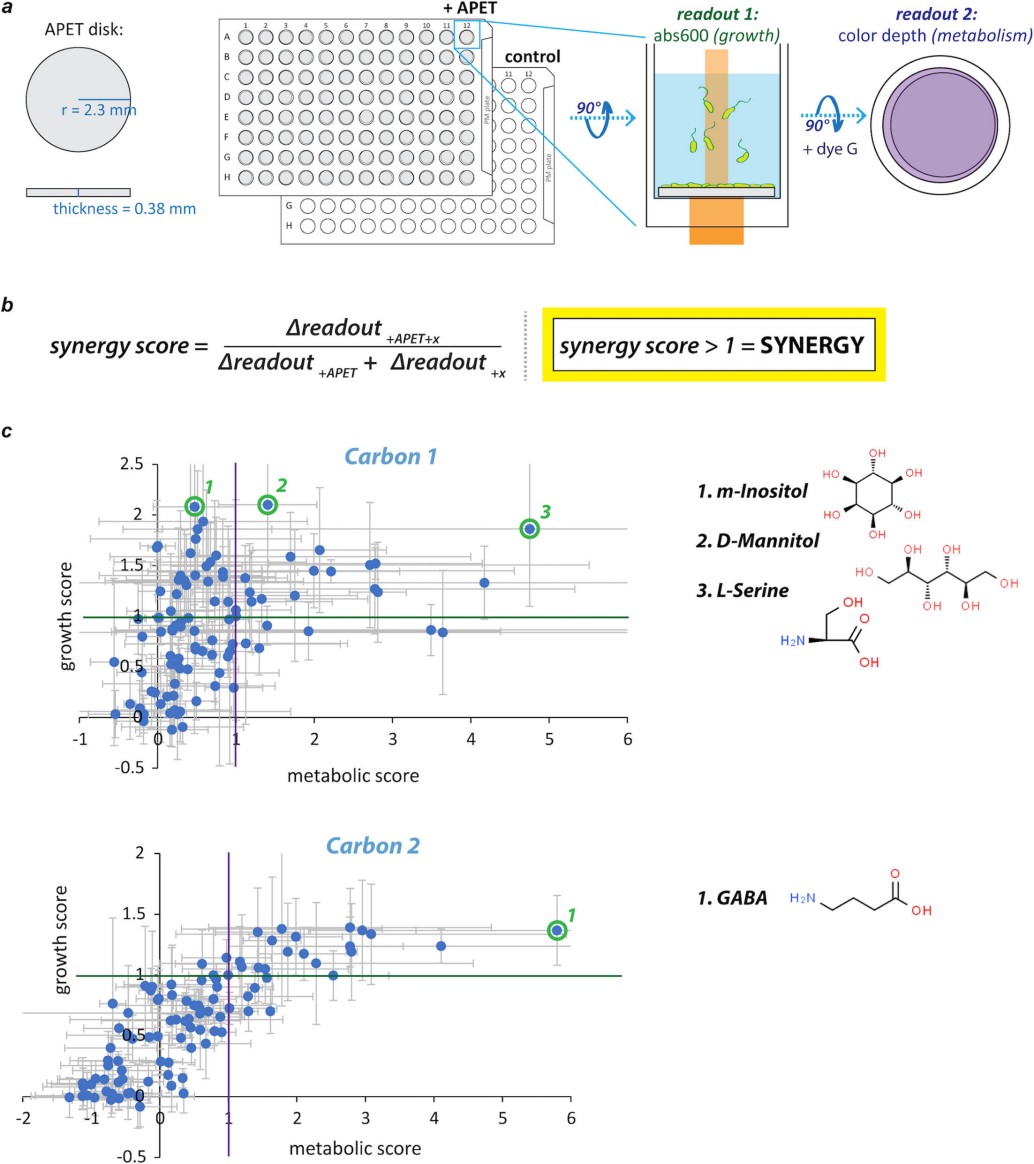
Screening for synergy or enhancement of PET-dependent growth and metabolism in phenotype microarrays. (**a**) A microplate-based assay for finding chemical conditions supporting enhanced PET-dependent growth and metabolism of *P. sakaiensis*. PM plates (Biolog) were prepared with and without disks of amorphous polyethylene terephthalate (PET), loaded with *P. sakaiensis* in YSV minimal medium, and incubated at 30°C with shaking for 4 days. Bacterial growth measurements were made every hour or once at the end of the experiment by recording A600s from each well. Metabolic activity was measured at a single time-point following the introduction of a redox dye (Biolog Redox Dye Mix G) on day 4 and a 24 h incubation. (**b**) Synergy scores. The two readouts were used to calculate synergy scores reflecting the presence (>1) or absence (≤1) of enhanced PET-dependent growth and/or metabolic activity (see Materials and Methods). Enhancements of either, but especially growth, were hypothesized to promote enhanced PET biodegradation. (**c**) Carbon source plates (PM1 and PM2A) showed correlated growth and metabolic scores, with a minority of conditions supporting both growth and metabolic synergy. Each plot contains 96 data points (95 test conditions + 1 no chemical control per PM plate) where each data point represents the mean of three independent experiments (error bars = standard deviation). Carbon 1 = PM1 plate, Carbon 2 = PM2A plate.

Further hypothesizing that *P. sakaiensis* can be readily screened for novel activators of PET biodegradation, we used Biolog phenotype microarray (PM) plates to search chemical/nutrient space for conditions stimulating PET-dependent bioactivity, with bacterial growth and metabolism as readouts (Fig. 1a). PET-dependent bacterial growth can report directly on the conversion of PET to microbial biomass, and PET-dependent metabolic activity, measured by adding a redox dye to the PM microcultures and recording the resultant color depth after a 24 h incubation, should report on the level of work performed by *P. sakaiensis*, a quantity that may be associated with elevated biodegradation. Each aspect of PET-dependent bioactivity was quantified using a synergy score, with values in excess of one indicating a non-linear increase in bacterial growth or metabolism from the relevant chemical:PET combination (Fig. 1b) (see Materials and Methods).

**Fig 2:**
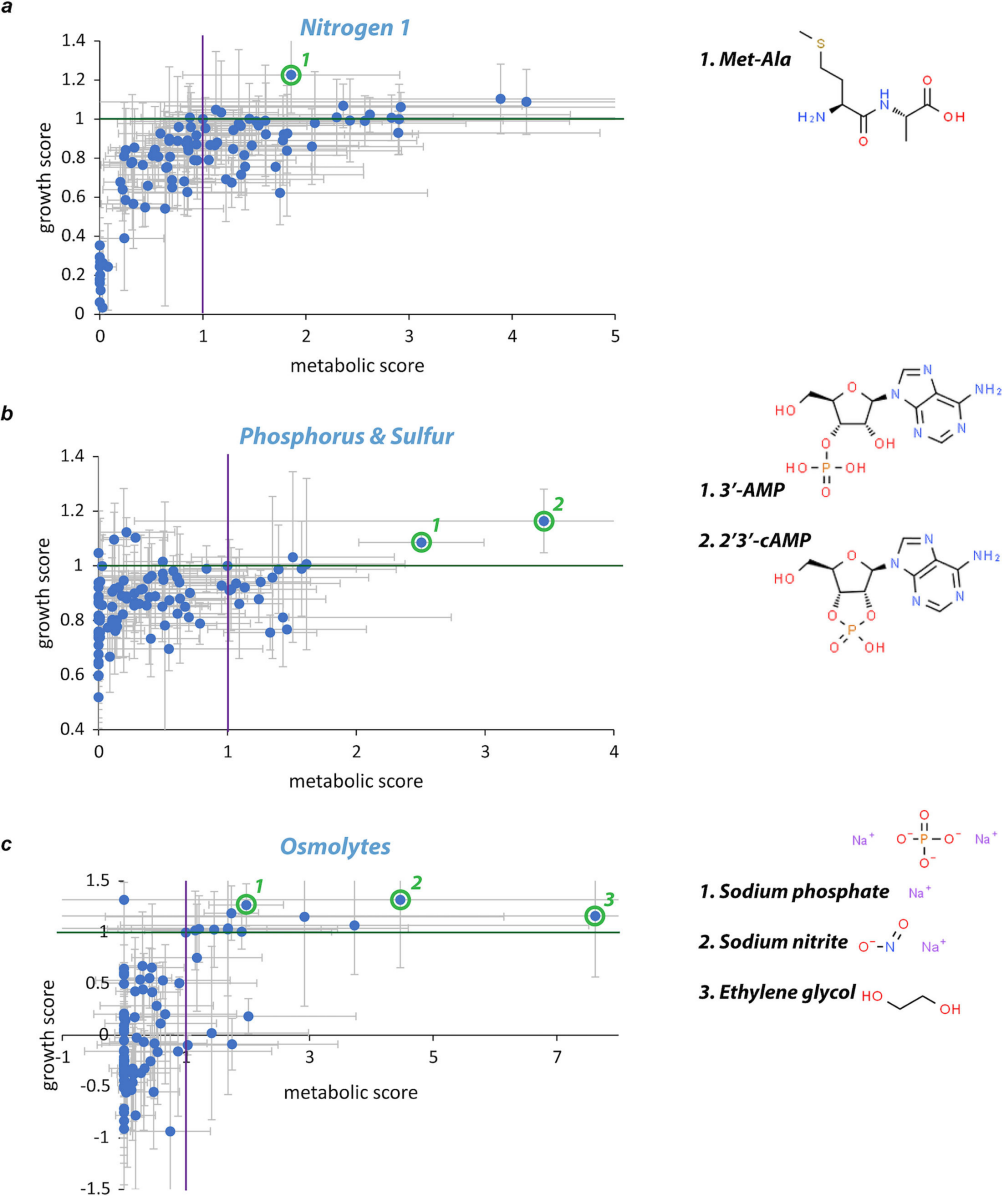
Few nutrient sources and inorganic compounds support both growth and metabolic synergies. (**a**) Nitrogen sources (PM3B) showed little synergy, with a dipeptide (Met-Ala) modestly boosting PET-dependent growth and metabolism. (**b**) Phosphorus and sulfur sources (PM4A): two nucleotide derivatives supported growth and metabolic synergy. (**c**) Osmolytes (PM9): two inorganic compounds and one byproduct of *P. sakaiensis* IsPETase activity supported growth and metabolic synergy. Each plot contains 96 data points (95 test conditions + 1 no chemical control) where each data point represents the mean of three independent experiments (error bars = standard deviation).

The full spectrum of possible bioactivity levels can be decomposed into four major quadrants (Fig. 1c and 2): (i) upper right conditions (*g* and *m* scores greater than one) support enhanced PET-dependent bacterial growth and metabolism, (ii) upper left conditions (only *g* scores greater than one) support greater than expected growth but less than expected metabolic activity, (iii) lower left conditions (*g* and *m* scores less than one) support diminished growth and metabolic activity, and (iv) lower right conditions (only *g* scores less than one) support diminished growth with greater than expected metabolic activity. Only the upper quadrants are potentially supportive of enhanced PET consumption, so we focused on “hits” from within these two regions. Moreover, we prioritized hits available for ≤$1 per gram for further study.

### Searching for hit concentrations supporting enhanced PET-dependent growth

**Fig 3:**
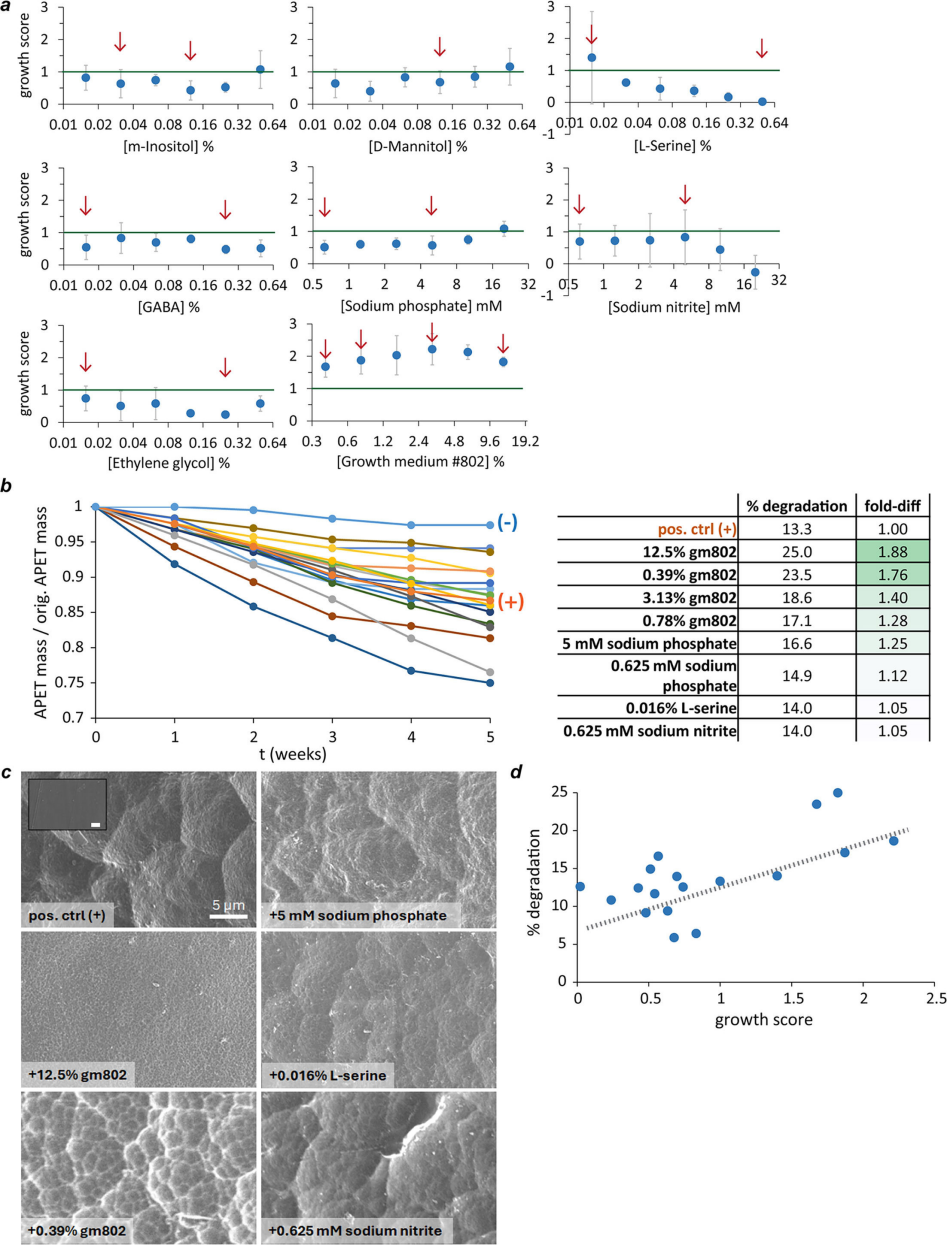
Higher growth scores correlate with faster PET biodegradation. (**a**) Growth scores over a range of concentrations of PM screen-identified hits and dilutions of growth medium #802 (gm802). Chemical conditions were prepared across a 32-fold range of concentrations and assayed in 24-well plates for growth scores after 2 days. Each data point represents the mean of three independent experiments (error bars = standard deviation). Data points marked with a red arrow indicate conditions chosen for a downstream PET biodegradation assay. (**b**) Dilutions of growth medium #802 and three other chemicals improved PET biodegradation. A 5-week-long PET biodegradation assay (see Materials and Methods) was performed using 17 test conditions and two unsupplemented YSV control conditions ([+] = with *P*. *sakaiensis* and [−] = no bacteria). PET strip masses were measured weekly. Fold differences in % biodegradation after 5 weeks (fold-diff) were calculated relative to the unsupplemented positive control. (**c**) Scanning electron microscopy (SEM) data showing nutrient-enhanced PET biodegradation. Representative SEM micrographs (scale bar = 5 µm) of biodegraded PET under a subset of conditions from biodegradation assay 1. Inset of undegraded PET. (**d**) The extent of PET biodegradation after 5 weeks correlated with the growth scores measured after 2 days (linear fit: *y* = 5.48*x* + 8.80, *R*^2^ = 0.45).

Chemical quantities in PM plates were not given, so it was necessary to determine the concentrations of hits supporting enhanced PET-dependent bioactivity. In addition, anticipating the need to scale up from 150 μL cultures to larger volumes needed for weeks-long PET biodegradation assays, we assayed bacterial growth scores across a 32-fold range of hit concentrations in a larger 24-well format (1.05 mL per well). Absorbance measurements were made after 2 days instead of 4 days because of enhanced evaporative loss likely due to increased surface wave action and fluid movement in the larger wells. Finally, we included dilutions of a rich culture medium (growth medium #802 or gm802) as an eighth possible hit. Using these conditions and scoring growth as described above, only 7 of 48 conditions assayed supported synergy scores greater than one (Fig. 3a). The six highest growth scores (range: 1.68–2.22) all came from dilutions of gm802, with the seventh highest coming from the lowest percentage (0.016%) of L-serine assayed (Fig. 3a).

### Assaying hits for enhanced PET biodegradation

We chose a subset of 17 conditions including high and low growth score extremes and positive and negative controls to scale up for PET biodegradation assays needed to determine the effectiveness of our bioactivity-based screening. Positive and negative controls contained unsupplemented yeast extract-sodium carbonate-vitamins (YSV) medium and were either inoculated with *P. sakaiensis* at the start of the experiment (positive control) or not (negative control). Larger cultures (4 mL) were used to accommodate plastic strips large enough for accurate weekly measurements of PET mass loss. Each culture contained two strips of amorphous PET plastic differing only slightly in shape (straight vs round edges) at a total loading ratio of approximately 2.5 cm^−1^ (loading ratio = initial PET surface area/total reaction volume). Every week, one strip was removed and processed (1% SDS wash, 70% ethanol wash, drying) before being weighed, and 90%–95% of the media was replaced with fresh media. The purpose of this strategy was to minimize the disruption of biodegradation by ensuring that each culture could always contain a population of *P. sakaiensis* in a PET-consuming biofilm. Biodegradation rates (mg cm^−2^ day^−1^) were measured from the weekly change in mass and used to estimate the total mass loss in each of the conditions assayed (Fig. 3b). A sample subset was subjected to scanning electron microscopy (SEM) to visualize biodegradation-induced changes to PET after 5 weeks (Fig. 3c).

Remarkably, the extent of biodegradation measured after 5 weeks correlated with the average growth scores measured after 2 days (Fig. 3d). An average PET biodegradation rate of 0.096 ± 0.037 mg cm^2^ day^−1^ was measured for the positive control (YSV only) (Table S1), which is ~50% of that reported by Yoshida et al. (19). A total of eight conditions supported equal or greater biodegradation than control, with the greatest gains coming from the four dilutions of gm802 tested (Fig. 3B). The two conditions showing the greatest enhancement were the high and low concentration extremes of gm802 (+88% and +76% from 12.5% and 0.39% gm802, respectively), but the biodegradation rate from 12.5% gm802 slowed markedly between weeks 4 and 5, while that of 0.39% showed no such slowing (Fig. 3b; Table S1). Conditions showing modestly enhanced biodegradation relative to control were 5 and 0.625 mM sodium phosphate pH 7 and 0.016% L-serine.

### Combining hits in search of greater enhancement of PET-dependent growth and biodegradation

**Fig 4:**
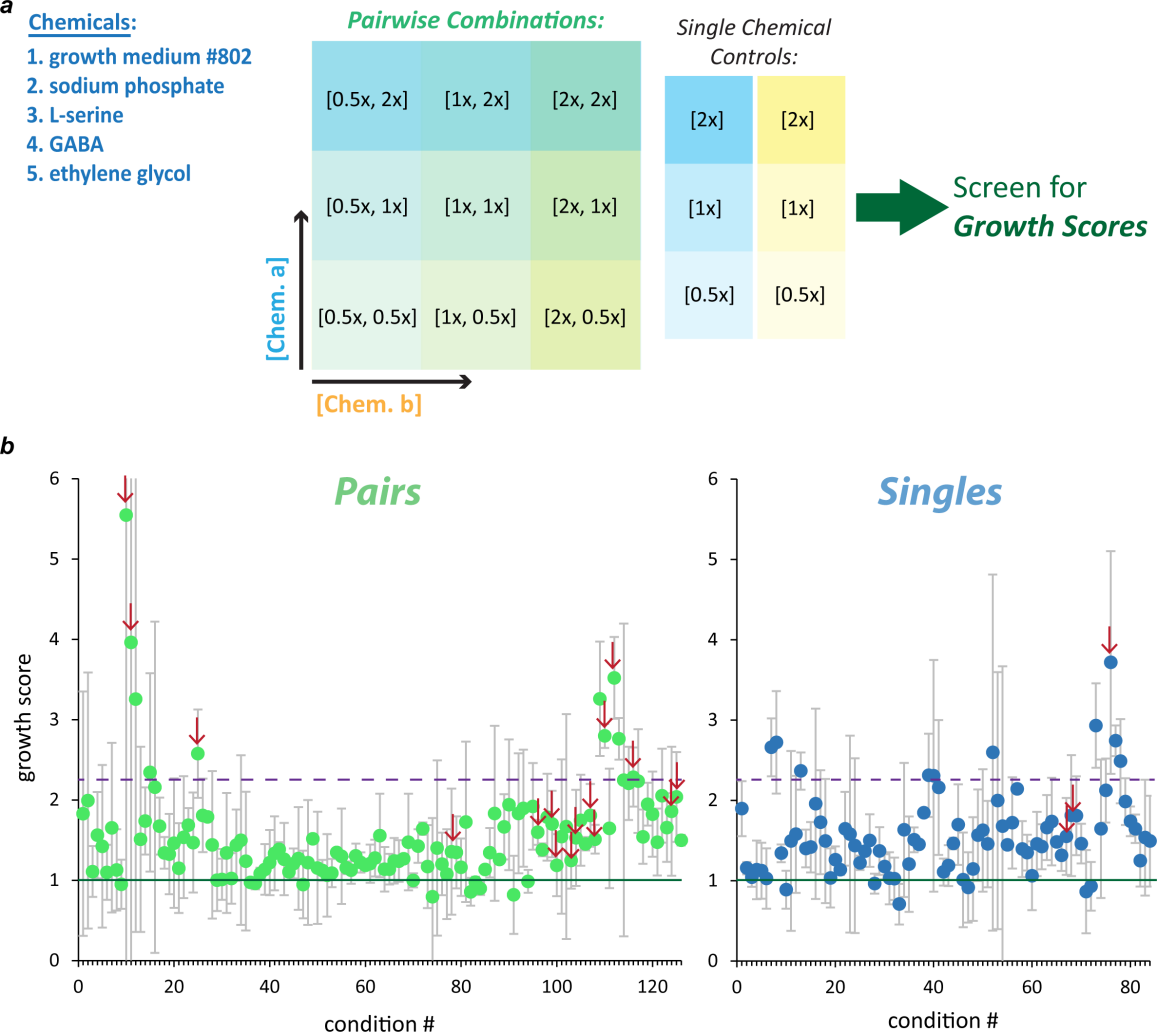
Screening pairwise combinations of choice chemicals for greater growth synergy. (**a**) Subset of hits chosen for screen of pairwise combinations. Five different chemical conditions were chosen for 14 different pairwise combinations: two different starting concentrations of gm802 (12.5% and 0.39%) × four chemicals (sodium phosphate, L-serine, GABA, and ethylene glycol) plus all six pairwise combinations generated from the four non-gm802 conditions. The dilution scheme was chosen to explore concentrations equal to, twofold higher, and twofold lower than those supporting or nearly supporting growth synergy for the single chemicals involved. Single chemical controls were also included. (**b**) Growth scores across pairwise combinations and single chemical controls. The vast majority of conditions tested supported growth synergy (data points above green line) and 10 pairwise combinations + 9 single chemical controls showed growth synergy exceeding the maximum observed in the first set of single chemical 24-well-plate assays performed (data points above dashed purple line). Each data point represents the mean of two independent experiments (error bars = standard deviation). Data points marked with a red arrow indicate conditions chosen for a downstream PET biodegradation assay.

We hypothesized that greater gains in PET-dependent bacterial growth and PET biodegradation could be realized by combining chemical conditions (Fig. 4). To that end, we searched over a range of concentrations within 14 different pairwise combinations for novel conditions supporting growth scores greater than one (Fig. 4a). The 14 combinations came from two different starting concentrations of gm802 (12.5% and 0.39%) × four chemicals (sodium phosphate, L-serine, GABA, and ethylene glycol) plus all six pairwise combinations generated from the four non-gm802 conditions. The dilution scheme was chosen to explore concentrations equal to twofold higher and twofold lower than those supporting or nearly supporting growth synergy for the single chemicals involved (Fig. 4a). Single chemical controls were also included in the screen (Fig. 4a).

**Fig 5:**
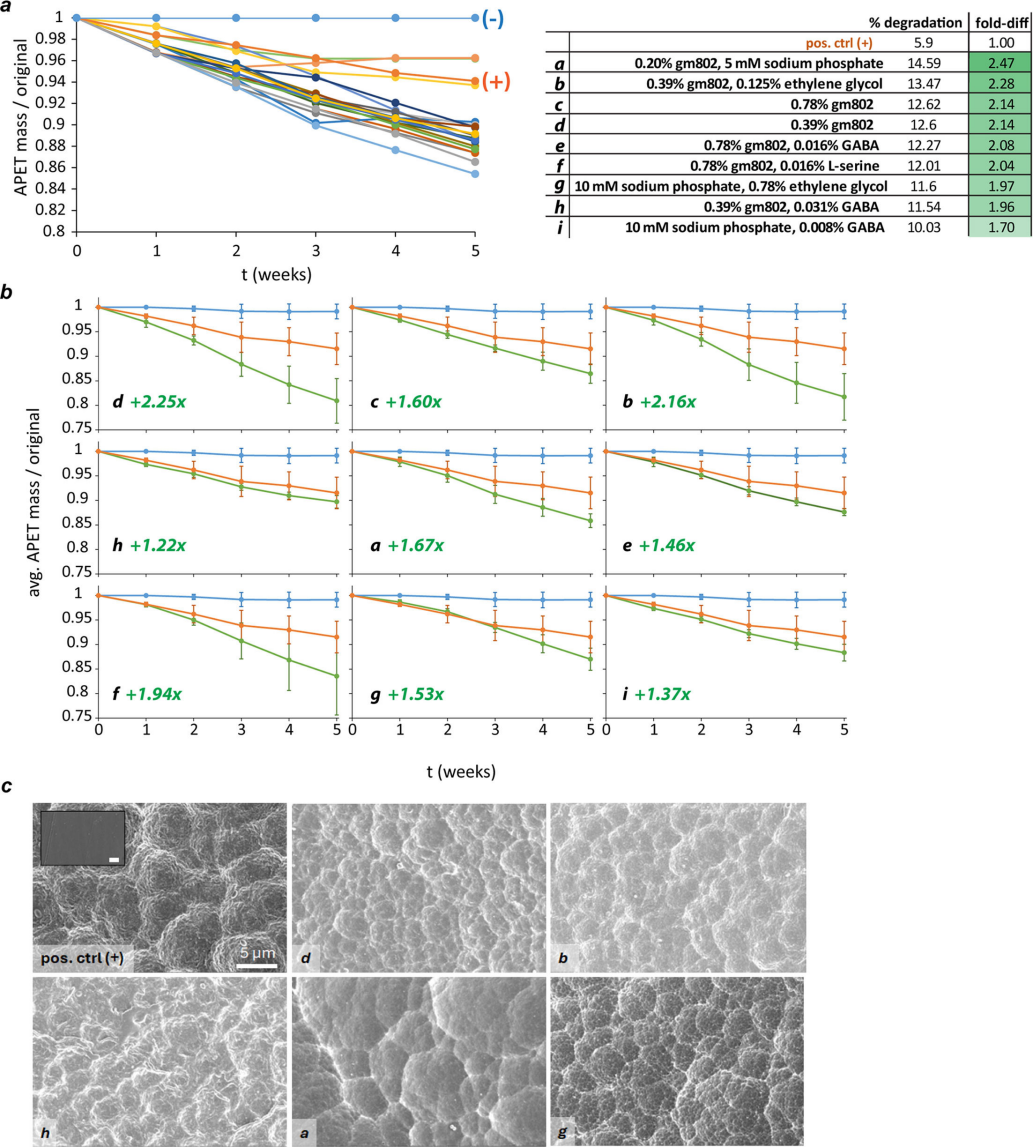
Highly dilute growth medium #802 and certain hit combos consistently enhance PET biodegradation. (**a**) A 5-week-long PET biodegradation assay (see Materials and Methods) was performed using 19 test conditions and two unsupplemented YSV control conditions ([+] = with *P*. *sakaiensis* and [−] = no bacteria). PET strip masses were measured weekly. Fold differences in % biodegradation after 5 weeks (fold-diff) were calculated relative to the unsupplemented positive control, with six conditions yielding twofold or higher enhancement. (**b**) Average degradation curves for nine enhancing conditions. A third biodegradation assay was performed using nine of the top conditions from assay 2 (in duplicate), and average PET biodegradation was calculated for each time-point from all assays performed (error bars = standard deviation). (**c**) SEM data showing nutrient-enhanced PET biodegradation. Representative SEM micrographs (scale bar = 5 µm) of biodegraded PET under a subset of conditions from biodegradation assays 2 and 3. Inset of undegraded PET.

Almost all growth scores were above one (92%), and 10 of the 126 pairwise combinations supported average growth scores higher than the maximum observed in the screen of single hits (Fig. 4b). Apart from exceptionally high growth scores from combinations of sodium phosphate and ethylene glycol, combinations involving dilutions of gm802 generally supported the highest growth scores. We selected a subset of 19 test conditions including three of the highest growth scores and three gm802 controls for another 5-week-long PET biodegradation assay (Fig. 5a; Table S2). The average PET biodegradation rate in unsupplemented YSV medium was 0.043 ± 0.011 mg cm^2^ day^−1^, approximately half that of the first experiment (Table S2). Almost all test conditions showed enhanced biodegradation relative to the positive control after 5 weeks (Fig. 5a; Table S2). A total of 10 conditions showed greater fold enhancement than the previous maximum of 1.88, with six conditions supporting greater than twofold enhancement (Fig. 5a). Most of the 17 conditions that supported enhanced biodegradation were dilutions of gm802 in combination with either sodium phosphate, ethylene glycol, GABA, or L-serine, but three contained no rich medium at all: these were sodium phosphate with either ethylene glycol (two conditions) or GABA. Consistent with assay 1, the biodegradation rate from 12.5% gm802 strongly decreased over the course of the experiment (Tables S1 and S2). In contrast, biodegradation rates from both combinations of sodium phosphate and ethylene glycol strongly increased over the 5 weeks assayed (Table S2).

Because of the noticeable change in control degradation rates between PET biodegradation assays 1 and 2, we decided to run a third 5 week biodegradation assay incorporating the top eight conditions from assay 2 along with 10 mM sodium phosphate + 0.01% GABA (#15 from assay 2, fold-diff = 1.70) and unsupplemented YSV controls (positive and negative). In addition, all conditions were prepared in duplicate. Merging and averaging across the weekly rates measured for the two positive controls yielded an average PET biodegradation rate of 0.052 ± 0.016 mg cm^2^ day^−1^, which is highly comparable to that measured for the single control from assay 2 and likewise ~50% of the relevant value from assay 1 (Tables S1 to S3). As expected from assay 2, all test conditions showed enhanced biodegradation after 5 weeks relative to the positive controls (Table S3). Intra-condition variation was generally low, with eight of the nine pairs showing comparable biodegradation rates between duplicates (Table S3). One set of duplicates showed high variation in the extent of PET biodegradation: *P. sakaiensis* growing in 0.78% gm802 + 0.016% L-serine degraded either 26% or 12% of amorphous PET after five weeks (Table S3). The source of this variation is unclear, but it began to emerge two weeks after the start of the assay and reached a maximum after four weeks. Although this magnitude of variation was seen for a single condition only, it made clear the need to average all data, where possible, across the three biodegradation assays performed (Fig. 5b). Doing so made it apparent that 0.39% gm802 and separately 0.39% gm802 + 0.125% ethylene glycol were two of the hit conditions with biodegradation-boosting effects most robustly supported by our data (Fig. 5b). Finally, a sample subset was subjected to SEM to visualize biodegradation-induced changes to PET after five weeks (Fig. 5c).

We also sought to confirm whether boosting PET biodegradation also boosted conversion to microbial biomass by comparing biodegradation rates with culture media visible light absorbance readings taken from the same cultures and time-points (Fig. S2). There is a strong correlation between the two, consistent with the known ability of *P. sakaiensis* to completely consume and metabolize PET.

### Testing the PET loading ratio for an effect on biodegradation

We incorporated another set of test conditions within our third biodegradation assay to preliminarily test the effect of decreasing the PET loading ratio on weekly biodegradation rates (Fig. S3). The chemical conditions were identical to those done in duplicate, but these were done singly and at twofold lower PET loading ratio (1.23 cm^−1^). All showed greater PET biodegradation than their cognate cultures with higher loading ratios, suggesting the importance of this parameter in optimizing PET bioconversion by *P. sakaiensis*. The gain in average weekly biodegradation rates ranged from 1.2-fold to 3.1-fold, with an average gain of 1.9-fold (Fig. S3).

## DISCUSSION

We report a novel strategy to search chemical/nutrient space for small molecules, compounds, and mixtures stimulating enhanced PET biodegradation by the PET consumer, *P. sakaiensis*. By employing PET-dependent bioactivity screening with bacterial growth and metabolic activity as readouts, we were able to identify a small set of chemical species and one complex mixture out of hundreds that synergistically enhanced *P. sakaiensis* bioactivity in the presence of amorphous PET. Downstream screening and multiple PET biodegradation assays revealed the exact conditions necessary to accelerate PET biodegradation, with a 0.39% dilution of growth medium #802 (gm802) alone and in combination with 0.125% ethylene glycol, on average more than doubling the extent of biodegradation. This work demarcates a chemical/nutrient subspace worth further exploring for enhanced PET biodegradation by *P. sakaiensis*. In addition to gm802 and ethylene glycol, notable chemical species are sodium phosphate, L-serine, and GABA. All are low-cost components with a high probability of being included in a novel specialized culture medium designed to promote enhanced PET biodegradation and consumption by *P. sakaiensis*.

It is worth revisiting the basic logic underlying our approach to begin understanding how the chemical conditions discovered might enhance PET biodegradation. An extrinsic factor (small molecule, salt, or mixture of compounds) can in principle stimulate enhanced PET biodegradation by directly or indirectly altering the expression, activity, or regulation of the PETase. To more clearly picture the possibilities, we must imagine the relevant sequence of cellular and molecular events. *P. sakaiensis* must first find and bind to PET. How and whether *P. sakaiensis* senses bulk PET remain unknown, but it may involve sensing a combination of surface mechanical and chemical properties. Once bound, *P. sakaiensis* forms a biofilm from which it secretes the PETase, either directly into the surrounding medium or down tubular appendages that may function like nanoscale degradation straws (19). Some aspects or consequences of these early events result in the upregulation of PETase transcription (19). The major product of PETase action is MHET (19), essentially a single TA monomer bound to an EG monomer by an ester linkage. As soon as MHET is produced and released, it can either (i) diffuse into the surrounding medium or (ii) get imported into the periplasmic space where it is cleaved into TA and EG by an MHETase. TA and EG are then transported to the cytoplasm and further metabolized (19, 25, 26, 28). As the concentration of MHET in the surrounding medium increases, it can bind and inhibit free PETases (40, 41). Indeed, any extrinsic factor affecting the rate of MHET buildup in the surrounding medium might alter the rate of PET biodegradation. More specifically, added chemicals or nutrients could decrease extracellular MHET and enhance PET biodegradation by, for example, stimulating expression of the outer membrane porins responsible for periplasmic import of MHET. The stimulatory effect of low concentrations of gm802 might partly work this way, while also supporting a more vigorous response to PET through heightened expression of other PET biodegradation and PET metabolism-related genes, including the PETase and MHETase genes.

Our data suggest that robust enhancement of PET biodegradation occurs only from concentrations of gm802 less than and likely considerably lower than 12.5% (Tables S1 and S2). At 12.5% gm802, PET biodegradation was strongly enhanced early on but showed decreasing weekly enhancement, especially after 3–4 weeks (Tables S1 and S2). At greater than or equal to 25% gm802, we have observed no enhancement, and at 100% gm802, we have observed no degradation at all (data not shown).

In contrast to the delayed negative effect of 12.5% gm802, the combination of 0.78% EG and 10 mM sodium phosphate generally showed an increase in PET biodegradation over the first 1–3 weeks assayed (Tables S2 and S3; Fig. S2). Negative feedback onto PETase activity from EG has been described for *in vitro* conditions (40), but the positive effect of this combination, and others involving EG, suggests a cellular mechanism coupling the sensing of EG with increased PETase expression. We hypothesize that this effect is enhanced, like the subtle effects of low levels of other stimulating chemical species identified here (L-serine, GABA, and sodium phosphate), when combined with a low percentage of gm802.

To our knowledge, our work is the first to systematically explore the effects of culture medium composition on PET biodegradation by *P. sakaiensis*. However, other groups have also reported PET biodegradation rates from unsupplemented YSV (19, 42), which we used as our control condition. Across the total of four replicates from three separate biodegradation assays, we measured an average weekly PET biodegradation rate of 0.061 ± 0.030 mg cm^−2^ day^−1^ from unsupplemented YSV. A value of 0.22 mg cm^−2^ day^−1^ can be gleaned from the data of Yoshida et al. (19) for the same condition. Although they report data from a single experiment only, the difference appears significant. In both studies, *P. sakaiensis* was grown with amorphous PET at 30°C and with comparable agitation and weekly replacement of culture media. However, Yoshida et al. did not report the culture volume and therefore the PET loading ratio is unknown. We define the PET loading ratio as the initial surface area of PET to the total culture volume. This value is related to the speed with which concentration-dependent feedback from extracellular byproducts of PET biodegradation (such as MHET), metabolism, and bacterial growth can affect the rate of PET biodegradation. Quite simply, a decreased PET loading ratio might enhance biodegradation by promoting more sustained and continuous PETase action via greater dilution of MHET. The PET loading ratio in most of our biodegradation assays was 2.5 cm^−1^ (~9.9 cm^2^ amorphous PET to 4 mL). When we decreased this value by a factor of two, PET biodegradation rates increased (Fig. S2). Thus, we suspect the PET loading ratio was closer to and even lower than 1.3 cm^−1^ in the experiment of Yoshida et al. Indeed, Walter et al. reported data indicating an even higher biodegradation rate of 0.65 mg cm^−2^ day^−1^ from disks of low-crystallinity waste PET and *P. sakaiensis* in unsupplemented YSV at the very low PET loading ratio of 0.07 cm^−1^ (42). All of these indicate the potential importance of the loading ratio and other environmental variables in (i) properly interpreting published data and (ii) developing an optimized fermentation process for the rapid conversion of PET to biomass.

Regarding the latter goal, and assuming a further doubling of the Walter et al. rate from the addition of the stimulators described here, one can reasonably imagine a continuous feed process able to completely ferment appreciable quantities of low-crystallinity PET at near ambient temperature in 3–4 weeks—the time it takes to make certain ales and other lighter varieties of beer. Such a process deployed at the scale of global beer production could result in the annual conversion of 10 s of megatons of processed PET waste into biomass for use in various eco-centric and green technological applications—this is the meaning of eco-microbiology.

Our study, like any, has obvious limitations. Gene expression studies are needed to decipher how low concentrations of gm802 (alone and in combination with low concentrations of other stimulators) and other stimulating conditions (e.g., 0.78% ethylene glycol + 10 mM sodium phosphate) affect enhanced PET biodegradation and consumption by *P. sakaiensis*. Additional work is also needed to confidently identify maximally enhancing conditions from within the spectrum of chemical species and concentrations afforded by the set of stimulators discovered here. To that end, more rapid and compact biodegradation assays might be ideal. Finally, all our experiments involved amorphous PET, so the potential effects of enhancing conditions on the biodegradation of more crystalline PET, including various forms of raw waste PET, remain unknown.

In conclusion, we took an innovative screening approach to sift through hundreds of chemical conditions for a select few able to enhance PET biodegradation by the naturally evolved PET consumer, *P. sakaiensis*. We measured slightly more than a doubling in the rate of amorphous PET biodegradation in the presence of low concentrations of gm802, both alone and in combination with other stimulators (ethylene glycol, sodium phosphate, L-serine, and GABA). Our results allow for the formulation of an inexpensive culture medium designed to enhance whole-cell PET biodegradation and consumption. Coupled with existing methods of processing PET waste into low-crystallinity and high surface “feedstock”, and assuming further gains in the PET biodegradation rate via optimally engineered process conditions, the results presented here bring us closer to generating a *P. sakaiensis*-based fermentation process for the rapid conversion of PET waste to microbial biomass at scale.

## MATERIALS AND METHODS

### *Piscinibacter sakaiensis* cultivation

Lyophilized *Piscinibacter sakaiensis* 201-F6 was received from the Biological Resource Center (NBRC), Japan. The lyophilized bacteria were reconstituted according to NBRC’s instructions. An initial glycerol stock (25% glycerol) was made from freshly reconstituted *P. sakaiensis* and used to streak plates of 1.5% agar in growth medium #802. Plates were incubated at 30°C for 4–5 days before the appearance of single colonies.

A second glycerol stock of larger volume was made for all experiments described here in the following manner: a single colony was used to inoculate 4 mL of YSV containing a strip of PET, and another single colony was used to inoculate 4 mL of only YSV; these cultures were grown for 6 days at 30°C with shaking (260–280 rpm) and checked for PET-dependent growth by measuring and comparing their optical densities (OD600); after confirming PET-dependent growth, 1 mL was taken from the PET-containing culture and used to inoculate 25 mL of gm802 within a 125 mL Erlenmeyer flask which was then grown at 30°C with shaking (280 rpm); the gm802 culture was grown to an OD600 of 1 and glycerol stocked by mixing the culture 1:1 with 50% glycerol in water and storing at −80°C in 1 mL aliquots. This glycerol stock was subjected to whole-genome sequencing (see below).

For all experiments described here, *P. sakaiensis* “overnight” cultures were prepared by inoculating thawed glycerol stock (treated as a 30× stock) directly into gm802 (3–15 mL cultures) and incubating at 30°C with shaking (260–280 rpm) for approximately 24 h. After growing, an OD600 was measured, and an appropriate volume of culture was pelleted (1,080 × *g* for 10 min) and washed in YSV medium (2×) for use in screening or PET biodegradation assays. We settled on this procedure after observing considerable variation in the growth time needed for “overnight” cultures inoculated with single colonies from plates (1.5% agar in gm802). Whether fresh colony-containing plates were stored at 4°C or RT, single colonies lost the ability to support planktonic growth in rich media after 2–3 weeks.

### Medium preparation

YSV medium and growth medium #802 were prepared according to Yoshida et al. (19).

### Preparation, storage, and use of amorphous PET

Sheets of amorphous PET (11″ × 12″ × 0.015″) were ordered from Polymer Firms (Tyngsboro, MA, USA) and cut into disks or strips of variable size using a Cricut Maker craft cutter (Cricut, South Jordan, UT, USA). PET pieces were stored in 70% ethanol and at 4°C. Before each experiment, PET pieces were removed from 70% ethanol and dried on plastic weigh dishes inside a biosafety cabinet. PET pieces were then UV irradiated for 10 min. After UV irradiation, PET pieces were introduced one-by-one into Biolog PM plates (Biolog, Inc., Hayward, CA, USA), 24-well plates, or 15 mL conicals used in PET biodegradation assays.

### Biolog PM and 24-well-plate screening

For a single experiment, a pair of Biolog PM plates of the same type (PM1, PM2A, PM3B, PM4A, or PM9) was taken out of storage (4°C), opened in a biosafety cabinet, and allowed to come to RT. A small disk of dried and UV-irradiated PET (*d* = 0.458 cm) was added to each of the 96 wells in a single PM plate (“+PET plate”). One hundred microliters of *P. sakaiensis* in YSV at OD600 = 0.1 was added to each well of the two PM plates using either a single-channel repeat or multi-channel pipettor while being careful not to touch the sides or bottom of the wells. The PET disks in the “+PET plate” were plunged to the bottom of each well, rendering only one side of the disk accessible to *P. sakaiensis* for binding and biodegradation. The PET loading ratio was ~1.65 cm^−1^. Each plate was then covered with its lid and sealed with parafilm before being placed in a plate reader or a standard shaker-incubator at 30°C and 260–280 rpm for 4–5 days of growth.

Bacterial growth was monitored via light absorbance (abs600) and recorded every hour (if incubated in a plate reader) or at a single time-point at the end of day 4. A redox indicator (Biolog Redox Dye Mix G, 100×) was then added to each well, and plates were incubated for another 24 h at 30°C and 260–280 rpm (Biolog, Inc., Hayward, CA, USA). Metabolic activity was measured at the end of day 5 by photographing plates using a digital camera suspended over a light curtain and calculating the color depth in each well (highest avg. pixel intensity − avg. pixel intensity in well).

Larger PET disks (*d* = 1.13 cm) were used for downstream screening in 24-well plates. Dilutions of single or combined chemicals/conditions were made in each plate. Plates were closed and sealed with parafilm and left to grow for 2 days in a shaker-incubator at 30°C and 260–280 rpm. Bacterial growth was measured by light absorbance (abs600) at a single time-point at the end of day 2 from (i) total well volumes and (ii) 120 μL media samples taken from each well. The latter specifically reports on the planktonic growth of *P. sakaiensis*, while the former also includes the absorbance/scattering effects of the PET-bound biofilm and biodegradation-induced changes to PET surface texture and opacity.

### Calculating synergy: growth and metabolic activity scores

Growth and metabolic scores were calculated to discover conditions supporting synergy or enhancement of PET-dependent growth and metabolism using the following formula: synergy score *=* Δreadout_+__PET_*_+x_*/(Δreadout*_+_*_PET_ *+* Δreadout*_+x_*). Δreadout equals the difference in readout value between the experimental condition and the negative control of unsupplemented YSV; *+x* refers to a variable chemical.

Single time-point measurements were used for calculating both growth and metabolic scores. Multi-time-point growth data were also available for some experiments—in those cases, we used a time-normalized growth integral to calculate growth scores.

### PET biodegradation assays

Amorphous PET plastic strips were retrieved from 70% ethanol within a biosafety cabinet where they were UV irradiated (10 min) and allowed to dry. A pair of PET strips, where individual strips differed slightly in size (each approx. 1.25″ × 0.31″ × 0.015″ for PET loading ratio = 2.5 cm^−1^ and 0.62″ × 0.31″ × 0.015″ for PET loading ratio = 1.23 cm^−1^) and shape (round vs straight edges), was prepared for each condition to be tested. Each pair was submerged in 4 mL of supplemented or unsupplemented (positive and negative controls) YSV medium in a 15 mL conical and 20 μL of *P. sakaiensis* in YSV at OD600 = 10.05 was added for a final OD600 of 0.05. Cultures were incubated at 30°C with shaking (approx. 270–280 rpm). Every week starting 1 week after the start of the experiment and ending after 5 weeks, a different PET strip (round or straight edges) was taken from each culture and processed (30 min wash with shaking in 1% SDS, 30 min wash with shaking in 70% ethanol) and dried before being weighed using an electronic balance (max = 60 g, *d* = 0.1 mg). Weights were rounded to the nearest mg and recorded and used to calculate PET biodegradation rates.

### Scanning electron microscopy

Samples were carbon-coated using the SPI Module Carbon Coater with two runs of 3 s each. Samples were imaged using a Scanning Electron Microscope (FEI Helios NanoLab 660 DualBeam System) under high vacuum at 2 kV, 0.1 nA current, and a working distance of 5–6.5 mm to achieve optimal results and minimize electron beam damage. Images were collected using the Everhart–Thornley detector in secondary electrons mode.

### Whole-genome sequencing

DNA from a 24 h culture of *P. sakaiensis* in gm802 was purified using a bacterial DNA kit (Omega Bio-Tek) and prepared into a library for Illumina sequencing by the Alkek Center for Metagenomics and Microbiome Research (CMMR) and the Human Genome Sequencing Center (HGSC) at Baylor College of Medicine.

Sequences were assembled in Geneious Prime (Geneious Prime 2024.0.4) and annotated using RASTtk. Reads were trimmed to a quality score of Q20, and reads that were shorter than 30 bps were filtered out using BBDuk (version 38.84) from the BBMap suite.

## Data Availability

Data from PET-dependent bioactivity screening and PET biodegradation assays are provided in the form of main figures and supplemental figures and tables. The *Piscinibacter sakaiensis* whole-genome sequence has been deposited into NCBI with accession number JBFBPI000000000.

## SUPPLEMENTAL MATERIAL

Tables S1 to S3; Fig. S1 to S3 aem.02118-24-s0001.pdf Tables summarize PET biodegradation assays; figures summarize observed correlations and effect of decreased PET loading ratio.

ASM does not own the copyrights to Supplemental Material that may be linked to, or accessed through, an article. The authors have granted ASM a non-exclusive, world-wide license to publish the Supplemental Material files. Please contact the corresponding author directly for reuse.
