# Supplementary material for: Eco-microbiology: discovering biochemical enhancers of PET biodegradation by Piscinibacter sakaiensis
Source: Appl Environ Microbiol. 2025 Feb 24;91(3):e02118-24. doi: 10.1128/aem.02118-24 (PMC11921318; doi:10.1128/aem.02118-24)
Supplement: Tables S1 to S3; Fig. S1 to S3 — Tables summarize PET biodegradation assays; figures summarize observed correlations and effect of decreased PET loading ratio. [file aem.02118-24-s0001.pdf]

condition #	chem1	[chem1]	% biodegradation	fold-diff	<biodegradation rate>	stdev	fold-diff	weekly change in biodegradation rate (%)				
								1-2	2-3	3-4	4-5	sum
1	m-Inositol	0.13	12.43	0.93	0.090	0.020	0.94	33.14	-12.37	-18.49	-24.50	-22.23
2	m-Inositol	0.03	9.41	0.71	0.068	0.015	0.71	-23.92	-12.37	-14.41	57.28	6.58
3	D-Mannitol	0.13	5.89	0.44	0.042	0.040	0.44	-23.92	-12.37	-100.00	0.00	-136.29
4	L-Serine	0.50	12.62	0.95	0.091	0.009	0.94	14.12	-12.37	14.12	-21.36	-5.50
5	L-Serine	0.02	14.03	1.05	0.102	0.043	1.06	-0.15	25.19	-31.53	-68.54	-75.04
6	GABA	0.25	9.15	0.69	0.067	0.048	0.70	-28.68	22.68	-83.70	-5.63	-95.32
7	GABA	0.02	11.67	0.88	0.084	0.076	0.88	128.23	-48.88	-67.40	-100.00	-88.04
8	sodium phosphate	5.00	16.63	1.25	0.120	0.032	1.25	-14.41	75.26	-33.43	-19.11	8.30
9	sodium phosphate	0.63	14.93	1.12	0.108	0.014	1.13	-0.15	0.15	-28.68	32.12	3.44
10	sodium nitrite	5.00	6.41	0.48	0.046	0.017	0.48	-14.41	16.84	-71.47	183.11	114.07
11	sodium nitrite	0.63	13.96	1.05	0.100	0.013	1.04	14.12	-12.37	33.14	-5.63	29.25
12	ethylene glycol	0.25	10.83	0.81	0.077	0.067	0.81	156.76	-2.63	-77.18	-100.00	-23.05
13	ethylene glycol	0.02	12.56	0.94	0.090	0.010	0.94	14.12	2.23	-18.49	-5.63	-7.77
14	gm802	12.50	25.00	1.88	0.179	0.084	1.87	-25.82	-25.85	3.74	-62.25	-110.19
15	gm802	3.13	18.64	1.40	0.135	0.073	1.41	-10.34	-4.40	-71.47	25.83	-60.38
16	gm802	0.78	17.09	1.28	0.122	0.025	1.27	33.14	0.15	14.12	17.96	65.36
17	gm802	0.39	23.48	1.76	0.168	0.022	1.76	2.70	16.84	14.12	-13.49	20.17
18 (pos)	--		13.31	1.00	0.096	0.037	1.00	33.14	25.19	-42.94	-43.38	-28.00
19 (neg)	--		2.61	0.20	0.019	0.020	0.20					

Table S1. Summary of PET biodegradation assay 1. A PET biodegradation assay (see Methods) was performed using 17 test conditions and 2 unsupplemented YSV control conditions [(pos) = with *P. sakaiensis*; (neg) = no bacteria]. Chemical concentrations in each supplemented test condition are given as percentages (w/v) for all but sodium phosphate pH 7 and sodium nitrite (mM). PET strip masses were measured weekly for 5 weeks. The percent biodegradation is the total PET mass degraded by *P. sakaiensis* after 5 weeks. Fold-differences in % biodegradation

(fold-diff) were calculated relative to the unsupplemented positive control. An average biodegradation rate and standard deviation ($mg \cdot cm^{-2} \cdot day^{-1}$) was calculated from the weekly biodegradation rates for each condition. Finally, weekly changes in biodegradation rate were calculated and summed for the PET biodegradation dynamics supported by each condition.

condition #	chem1	[chem1]	chem2	[chem2]	% biodegradation	fold-diff	<biodegradation rate>	stdev	fold-diff	weekly change in biodegradation rate (%) 1-2	2-3	3-4	4-5	sum
**1**	gm802	0.39	--	--	12.60	2.13	0.093	0.023	2.15	-0.15	-24.89	-23.92	17.96	-31.00
**2**	gm802	0.78	--	--	12.62	2.14	0.093	0.018	2.14	-14.41	-12.37	-23.92	41.56	-9.15
**3**	gm802	12.50	--	--	9.74	1.65	0.070	0.068	1.61	33.14	25.19	-111.41	-194.37	-247.46
**4**	GABA	0.03	--	--	3.81	0.64	0.027	0.027	0.63	-14.41	-41.58	-100.00	0.00	-155.99
**5**	sodium phosphate	10.00	ethylene glycol	0.78	11.60	1.96	0.088	0.040	2.03	128.23	53.35	14.12	7.85	203.55
**6**	sodium phosphate	5.00	ethylene glycol	0.78	6.29	1.06	0.047	0.029	1.08	185.29	-12.37	-77.18	88.74	184.49
**7**	sodium phosphate	10.00	GABA	0.01	10.03	1.70	0.074	0.032	1.71	14.12	2.23	-67.40	41.56	-9.49
**8**	gm802	6.25	GABA	0.02	3.75	0.64	0.027	0.057	0.62	-57.21	-129.21	14.12	-100.00	-272.30
**9**	gm802	0.20	sodium phosphate	5.00	14.59	2.47	0.108	0.019	2.50	-0.15	12.67	-36.60	13.24	-10.84
**10**	gm802	0.20	sodium phosphate	2.50	11.27	1.91	0.084	0.018	1.93	14.12	2.23	-34.79	-5.63	-24.07
**11**	gm802	0.78	L-serine	0.03	10.24	1.73	0.075	0.035	1.72	-57.21	-41.58	185.29	13.24	99.75
**12**	gm802	0.78	L-serine	0.02	12.01	2.03	0.090	0.007	2.09	-4.90	5.16	14.12	-5.63	8.74
**13**	gm802	0.39	L-serine	0.02	11.00	1.86	0.083	0.020	1.92	-4.90	22.68	-51.09	88.74	55.43
**14**	gm802	0.39	L-serine	0.01	10.17	1.72	0.074	0.032	1.72	-28.68	-29.90	42.65	-62.25	-78.18
**15**	gm802	0.39	GABA	0.03	11.54	1.95	0.086	0.021	1.99	-23.92	75.26	-42.94	41.56	49.95
**16**	gm802	0.78	GABA	0.02	12.27	2.08	0.090	0.017	2.08	-28.68	-12.37	14.12	13.24	-13.69
**17**	gm802	0.20	GABA	0.01	11.42	1.93	0.083	0.021	1.92	-42.94	31.44	-23.92	17.96	-17.46
**18**	gm802	0.78	ethylene glycol	0.13	10.86	1.84	0.080	0.016	1.86	-4.90	22.68	-34.79	-5.63	-22.64
**19**	gm802	0.39	ethylene glycol	0.13	13.47	2.28	0.099	0.014	2.29	-14.41	-12.37	-4.90	32.12	0.43
**20 (pos)**	--		--		5.91	1.00	0.043	0.011	1.00	-42.94	31.44	14.12	-37.09	-34.47
**21 (neg)**	--		--		0.00		0.000	0.000						

Table S2. Summary of PET biodegradation assay 2. A PET biodegradation assay (see Methods) was performed using 19 test conditions and 2 unsupplemented YSV control conditions

[(pos) = with *P. sakaiensis*; (neg) = no bacteria]. Chemical concentrations in each supplemented test condition are given as percentages (w/v) for all but sodium phosphate pH 7 (mM). PET strip masses were measured weekly for 5 weeks. The percent biodegradation is the total PET mass degraded by *P. sakaiensis* after 5 weeks. Fold-differences in % biodegradation (fold-diff) were calculated relative to the unsupplemented positive control. An average biodegradation rate and standard deviation ($mg\cdot cm^{-2}\cdot day^{-1}$) was calculated from the weekly biodegradation rates for each condition. Finally, weekly changes in biodegradation rate were calculated and summed for the PET biodegradation dynamics supported by each condition.

condition #	chem1	[chem1]	chem2	[chem2]	% biodegradation	fold-diff	<biodegradation rate>	stdev	fold-diff	weekly change in biodegradation rate (%) 1-2	2-3	3-4	4-5	sum
**1a**	sodium phosphate	10.00	GABA	0.008	13.43	1.78	0.093	0.015	1.79	-30.33	43.52	-16.39	-10.30	-13.49
**1b**	sodium phosphate	10.00	GABA	0.008	11.52	1.53	0.080	0.015	1.52	-25.68	56.98	-4.45	-25.25	1.61
**2a**	gm802	0.39	GABA	0.031	10.33	1.37	0.071	0.030	1.36	-30.33	7.64	-25.68	-55.15	-103.51
**2b**	gm802	0.39	GABA	0.031	8.98	1.19	0.063	0.022	1.20	-25.68	34.55	-25.68	-55.15	-71.96
**3a**	sodium phosphate	10.00	ethylene glycol	0.780	11.76	1.56	0.080	0.017	1.52	39.35	25.58	-20.37	25.58	70.14
**3b**	sodium phosphate	10.00	ethylene glycol	0.780	15.58	2.07	0.105	0.045	2.02	11.48	124.26	11.48	-19.27	127.95
**4a**	gm802	0.78	L-serine	0.016	11.72	1.55	0.083	0.020	1.58	67.220	19.603	-30.325	7.643	64.141
**4b**	gm802	0.78	L-serine	0.016	25.59	3.39	0.172	0.075	3.29	178.70	61.46	-7.10	-22.26	210.81
**5a**	gm802	0.78	GABA	0.016	11.71	1.55	0.080	0.021	1.53	67.22	19.60	-30.33	-10.30	46.20
**5b**	gm802	0.78	GABA	0.016	13.14	1.74	0.091	0.038	1.74	95.09	40.96	-49.33	-10.30	76.43
**6a**	gm802	0.39	--		22.43	2.97	0.154	0.070	2.95	178.70	61.46	-25.68	-25.25	189.24
**6b**	gm802	0.39	--		19.27	2.56	0.137	0.023	2.62	11.48	12.13	22.63	-10.30	35.94
**7a**	gm802	0.78	--		12.92	1.71	0.091	0.011	1.74	11.48	19.60	-16.39	-10.30	4.40
**7b**	gm802	0.78	--		12.16	1.61	0.085	0.015	1.63	11.48	4.65	-36.30	56.98	36.81
**8a**	gm802	0.39	ethylene glycol	0.125	18.46	2.45	0.129	0.068	2.47	150.83	59.47	-23.36	-59.23	127.72
**8b**	gm802	0.39	ethylene glycol	0.125	22.93	3.04	0.156	0.047	2.99	53.28	38.63	-40.98	-0.33	50.60
**9a**	gm802	0.20	sodium phosphate	5.000	15.27	2.02	0.108	0.044	2.07	95.09	66.59	-39.97	-10.30	111.41
**9b**	gm802	0.20	sodium phosphate	5.000	12.56	1.67	0.085	0.021	1.63	39.35	25.58	-4.45	19.60	80.09
**10a (pos)**	--		--		8.76	1.16	0.059	0.014	1.14	11.48	34.55	-44.26	19.60	21.38
**10b (pos)**	--		--		6.32	0.84	0.045	0.017	0.86	-16.39	19.60	-72.13	258.81	189.89
**11 (neg)**	--		--		0.00		0.000	0.000						

Table S3. Summary of PET biodegradation assay 3. A PET biodegradation assay (see Methods) was performed using 9 test conditions and 2 unsupplemented YSV control conditions [(pos) = with *P. sakaiensis*; (neg) = no bacteria]. Conditions 1-10 were done in duplicate (a & b). Chemical concentrations in each supplemented test condition are given as percentages (w/v) for all but sodium phosphate pH 7 (mM). PET strip masses were measured weekly for 5 weeks. The

percent biodegradation is the total PET mass degraded by *P. sakaiensis* after 5 weeks. Fold-differences in % biodegradation (fold-diff) were calculated relative to the unsupplemented positive control. An average biodegradation rate and standard deviation ($mg \cdot cm^{-2} \cdot day^{-1}$) was calculated from the weekly biodegradation rates for each condition. Finally, weekly changes in biodegradation rate were calculated and summed for the PET biodegradation dynamics supported by each condition.

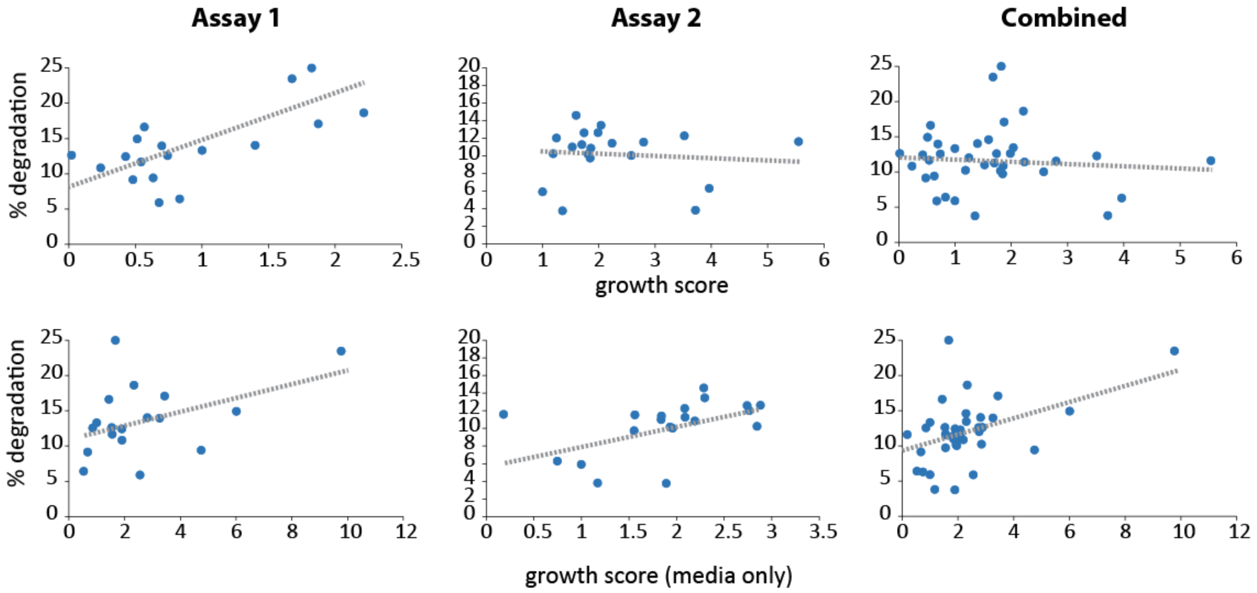

Fig S1. The extent of PET biodegradation after 5 weeks correlates with growth scores. Percent biodegradation after 5 weeks vs. growth scores measured after 2 days for biodegradation assay 1 (left plots), assay 2 (middle plots), and combined data (right plots). Two different growth scores were calculated: 1) from A600 measurements of each well of the 24-well plates (= growth score); and 2) from A600 measurements of 120 microL media taken from each well of the 24-well plates and transferred to a 96-well plate (= media only growth score). A600 measurements from media only are specific for planktonic bacterial growth, while A600 measurements from 24-well plates are affected by variable light scattering from partly degraded PET, the thickness of the PET-bound biofilm, and the presence of planktonic bacteria. (Linear fits, clockwise starting from the top left: 1) $y = 5.48x + 8.80$; $R^2 = 0.45$; 2) $y = -0.23x + 10.77$; $R^2 = 0.01$; 3) $y = 0.30x + 12.42$; $R^2 = 0.01$; 4) $y = 1.28x + 9.04$; $R^2 = 0.23$; 5) $y = 2.08x + 6.32$; $R^2 = 0.24$; and 6) $y = 0.98x + 11.18$; $R^2 = 0.19$).

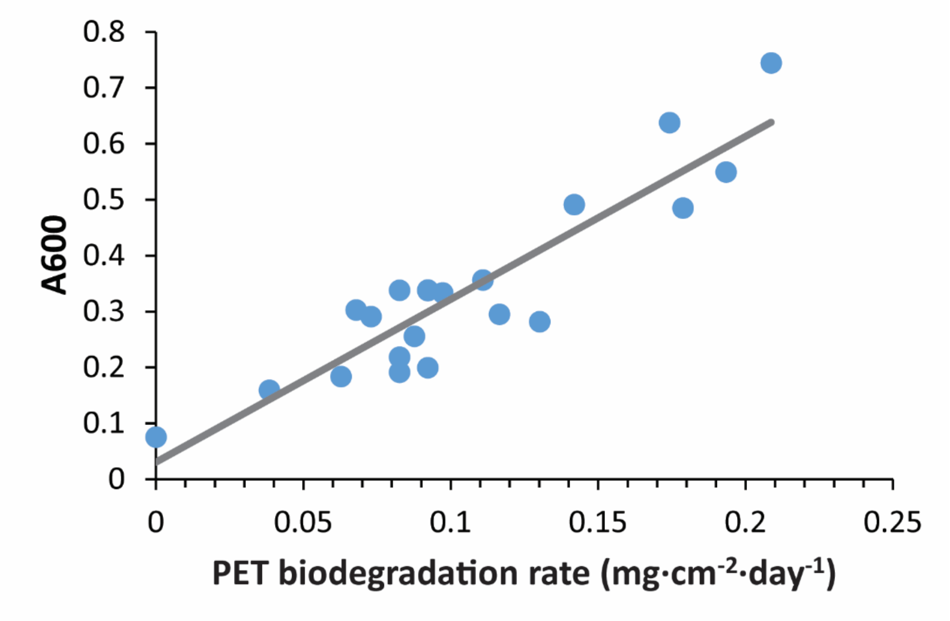

Fig S2. Microbial growth strongly correlates with PET biodegradation rates. Average weekly culture media A600 vs. average weekly PET biodegradation rate measured for biodegradation assay 3. Linear fit: $y = 2.91x + 0.03$; $R^2 = 0.83$

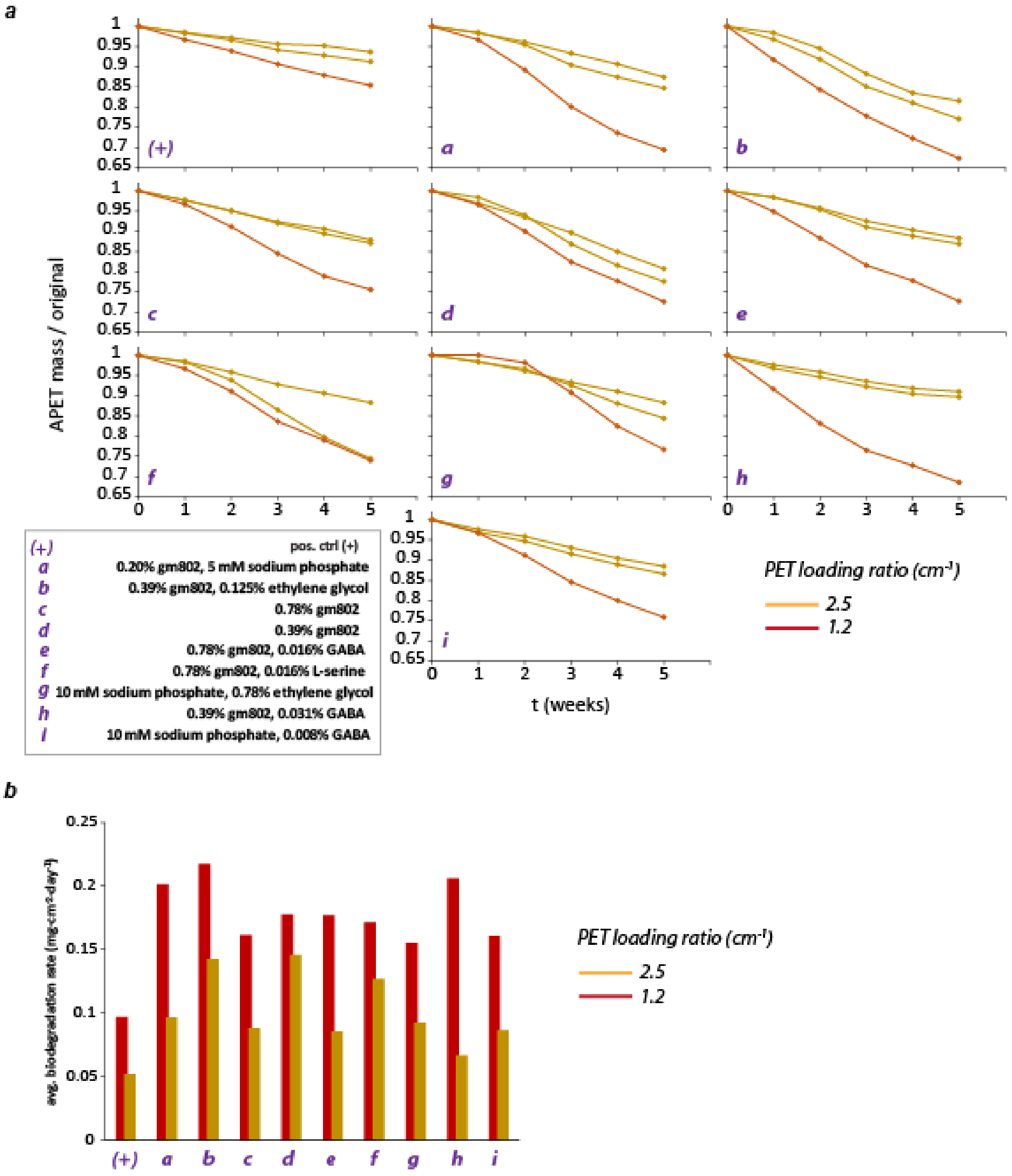

Fig S3. Lowering the PET loading ratio increases biodegradation by *P. sakaiensis*. (A) PET biodegradation plots showing total PET mass over time for 10 different culture conditions (*[+]* and *a-i*), each at two different PET loading ratios (2.5 cm^{-1} in yellow and 1.2cm^{-1} in red). Assays with PET loading ratio of 2.5 cm^{-1} were done in duplicate. **(B)** Average weekly PET biodegradation rates increase with decreasing PET loading ratio.
